# THE PERCEPTION OF CAREGIVERS OF POOR ORAL HEALTH OF THEIR CHILDREN AND ITS RELATED CLINICAL CONDITIONS

**DOI:** 10.1590/1984-0462/2021/39/2019381

**Published:** 2021-02-03

**Authors:** Jéssica Copetti Barasuol, Josiane Pezzini Soares, Michele Bolan, Mariane Cardoso

**Affiliations:** a Universidade Federal de Santa Catarina, Florianópolis, SC, Brazil.

**Keywords:** Oral health, Dental caries, Child, preschool, Open bite, Tooth fractures., Saúde bucal, Cárie dentária, Pré-escolar, Mordida aberta, Fraturas dos dentes

## Abstract

**Objective:**

To determine the association between the perception of caregivers regarding the oral health of their children and socio-demographic characteristics, report of dental pain, and clinical oral conditions.

**Methods:**

A cross-sectional study was conducted with 570 children aged two to five years old, enrolled at public preschools, and with their caregivers. Data regarding perceptions of oral health status in children, socio-demographic characteristics, and dental pain were collected from a questionnaire. Three examiners (*Kappa*>0.7) evaluated children’s oral health status using the dmft index, pufa index, and the Andreasen classification for traumatic dental injury (TDI). The occurrence of open bite and overjet was also investigated. Descriptive analyses, and unadjusted and adjusted logistic regression were used, considering a 5% significance level.

**Results:**

A total of 24.7% of children had poor oral health status, which increased 4.92-fold (95% confidence interval [95%CI] 3.05-7.93) when children had dental caries, and 3.78-fold (95%CI 1.63-8.76) when there were consequences from dental caries. The perception of poor oral health was also associated to open bite (*Odds Ratio* [OR] 1.98; 95%CI 1.16-3.38) and TDI (OR 1.68; 95%CI 1.06-2.68). No associations were found between the perception of caregivers and socio-demographic variables or overjet.

**Conclusions:**

The perception of caregivers of poor oral health in their children was associated to dental caries, its consequences, TDI, and open bite.

## RESUMO

**Objetivo:**

Determinar a associação entre a percepção dos cuidadores sobre a saúde bucal das crianças e características sociodemográficas, relato de dor dentária e condições clínicas bucais.

**Métodos:**

Foi realizado um estudo transversal com 570 crianças de dois a cinco anos matriculadas em pré-escolas públicas e seus cuidadores. Os dados referentes à percepção do estado de saúde bucal nas crianças, características sociodemográficas e dor dentária foram coletados por meio de um questionário. Três examinadores (*Kappa*>0,7) avaliaram o estado de saúde bucal das crianças usando o índice ceo-d, o índice PUFA e a classificação de Andreasen para traumatismo dentário (TD). A ocorrência de mordida aberta e *overjet* também foi investigada. Análise descritiva e regressão logística não ajustada e ajustada foram realizadas, considerando um nível de significância de 5%.

**Resultados:**

A percepção da má condição de saúde bucal nas crianças foi de 24,7%, a qual aumentou 4,92 vezes (IC95% ­3,05-7,93) quando as crianças apresentaram cárie dentária e 3,78 vezes (intervalo de confiança de 95% [IC95%] 1,63-8,76) quando houve consequências de cárie dentária. A percepção de saúde bucal ruim também foi associada a mordida aberta (*Odds Ratio* [OR] 1,98; IC95% 1,16-3,38) e TD (OR 1,68; IC95% 1,06-2,68). Não foram encontradas associações entre as percepções dos cuidadores e variáveis sociodemográficas ou *overjet*.

**Conclusões:**

As percepções dos cuidadores sobre a má condição de saúde bucal das crianças foram associadas à cárie dentária, suas consequências, TD e mordida aberta.

## INTRODUCTION

Many children are affected by oral problems, such as dental caries and their consequences, whose prevalence varies between 26^1^ and 69%,^2^ dental traumatic injuries, with prevalence between 33.5^3^ and 53.4%,^4^ and malocclusions, whose prevalence varies between 24.2^3^ and 63.3%.^5^ These oral problems are related to a negative impact on the oral health and its related quality of life of children, which, consequently, affect children’s adequate growth and development.^1^ ^,^ ^2^ ^,^ ^3^ ^,^ ^4^ ^,^ ^5^ Therefore, understanding and perceiving the oral condition of these children is essential for taking actions to regarding their prevention and treatment. Parents play an important role by ensuring that their children receive oral and medical health care,^6^ and their decisions affect children’s well-being and can determine whether pediatric dental treatment is sought.^7^ ^,^ ^8^

Among the problems that affect the oral cavity of children, early childhood caries (ECC) is a significant problem, defined as the presence of one or more decayed, filled, or missing teeth due to caries by the age of six.^9^ This is a problem of rapid progression, and parents often only notice an oral condition when their children complain of toothache or exhibit one of the major consequences of caries, requiring immediate action.^10^ ^,^ ^11^

Perception of oral health conditions requires knowledge of caregivers about alterations that can affect oral health, attention, and care for children.^2^ ^,^ ^5^ Besides that, socioeconomic factors, such as schooling and income, may also be related to this perception, and the appearance and progression of oral disease.^2^ ^,^ ^5^ Understanding which socioeconomic characteristics and clinical oral conditions of children are related to the perception of their caregivers can help professionals to create specific preventive actions. Providing information related to the importance of dental care for children is also important not only for treatment of established diseases but also to prevent them.

Thus, the present study aimed to determine associations between the perception of caregivers regarding the oral health of their children and socio-demographic characteristics, reports of dental pain in children, and clinical oral conditions. The null hypothesis is that there is no association between the perception of caregivers as to the oral health of their children and socio-demographic characteristics, reports of dental pain in children, and clinical oral conditions.

## METHOD

The study received the approval of the Research Ethics Committee of Universidade Federal de Santa Catarina (Protocol No.: 343.658), and it was conducted in compliance with the ethical standards defined in the Declaration of Helsinki. All caregivers signed an informed consent form authorizing their participation and that of their children.

A cross-sectional study was conducted with preschool children aged two to five years old and with their caregivers. Data were collected at public preschools in Florianópolis City, Brazil. According to the Brazilian Education Ministry, the city has 85 public preschools, in which 6,349 children are enrolled.^12^

Sample size was calculated with the aid of the G*Power program (v 3.1.9.2, University Kiel, Germany)^13^ using data from a pilot project in which the prevalence of the perception of caregivers of poor oral health in their children was 41% for children with dental caries and 28% for children with no caries. Considering binomial distribution, a 90% test power, a 5% significance level, and the addition of 20% to compensate for possible dropouts, a total sample of 676 pairs of caregivers and children was determined.

For selecting the sample, all preschools were invited to participate, and 46 authorized data collection. The students of these preschools who had the informed consent forms participated in a simple randomization process, considering the numbers in the children’s call list. The number of children selected to participate was proportional to the number of students enrolled in each school. Data were collected between March and December 2014.

The inclusion criteria were being from two to five years old, enrolled in a public preschool, being in the primary dentition phase or with mixed dentition, and having an informed consent form signed by a caregiver. Children with uncooperative behavior during the examination, with history of orthodontic treatment, and those who had any visually perceivable systemic or intellectual impairment reported by their guardians or teachers were excluded.

Pilot project was conducted with 20 children aged two to five, enrolled at a public preschool; methods were tested, and performed examiners’ calibration was performed. These individuals did not participate in the main study.

Calibration exercise involved a discussion and analysis of photographs, using the indexes to be employed in the main study for evaluating children’s oral health status. In the next step, the reference standard (a dentist previously calibrated for the use of these indexes) and three examiners evaluated 20 children. Clinical data were compared for the determination of the inter-examiner agreement with *Kappa*>0.75. After 14 days, the same children were evaluated a second time, and data were compared to those obtained during the first evaluation for the determination of intra-examiner agreement (*Kappa*>0.80).

Three calibrated examiners blinded to information on the caregivers’ responses performed the clinical examinations at the preschools. Each child was instructed to brush their teeth and was then seated on a chair. Visual inspection of the oral cavity was performed with the aid of artificial light (light-emitting diode) (Zeiss, Oberkochen, Germany) and #5 mouth mirror (Golgran, São Caetano do Sul City, Brazil).

Traumatic dental injury was investigated with the criteria proposed by Andreasen et al.^14^ and dichotomized as present (enamel fracture, enamel-dentin fracture, crown discoloration, and avulsion) or absent. Anterior open bite was defined as the absence of vertical overlap of the mandibular incisors by the maxillary incisors and classified as present or absent.^1^ Overjet was also classified as present (≥3 mm) or absent.^15^

Caries experience was determined using the decayed, missed, and filled teeth (dmft) index, proposed by the World Health Organization,^16^ and was dichotomized as present (≥1 decayed, missed, filled tooth) or absent (all sound teeth). The consequences of dental caries were measured using the pufa index and classified as present (≥1 primary tooth with pulp involvement, ulceration, fistula or abscess) or absent (without signs).^17^

A questionnaire was sent for caregivers to answer at home. Dependent variable was collected based on the answer to the question: “What do you think about the oral health status of your child?”. Responses were dichotomized as good (response options: very good and good) and poor (response options: fair, poor, and very poor). Dental pain was investigated based on the answer to the following question: “Has your child ever had a toothache?” (Yes or No).

The following socio-demographic data were collected: child’s sex and age, caregiver’s schooling (dichotomized >8 years, or ≤8 years), household income classified according to Brazilian Association of Research Companies (Associação Brasileira de Empresas de Pesquisa)^17^ (class A - 46 to 35 points or US$ ­2,526-1,703; class B - 34 to 23 points or US$ 903-522; classes C and D - 22 to 8 points or US$ 310-125).

Statistical analysis was performed with the *Statistical Package for the Social Sciences* (SPSS), version 21 (SPSS Inc, Chicago, IL, USA). Descriptive analyzes and logistic regression were used to determine relations between independent variables and the perception of caregivers regarding the oral health status of their children. An unadjusted logistic regression model was used to determine associations between dependent and independent variables. Independent variables with p<0.20 were incorporated into the adjusted model using the “enter” method. *Odds Ratios* (ORs) and respective 95% confidence intervals (95%CIs) were calculated considering a 5% significance level.

## RESULTS

A total of 676 preschools were potentially eligible, but only 570 participated in the study. Response rate was 84.3%. Missing data were due to refusals to participate (n=26), and unanswered questionnaires (n=80). Table 1 displays the sample characterization. Most children were between four and five years of age. According to the perception of caregivers, 141 children had poor oral health (24.7%).

**Table 1:** Descriptive analysis of caregivers’ perceptions of oral health in their children and independent variables. Florianopolis, Brazil (n=570).

Caregivers’ oral health perception of children		
	Good n (%)	Poor n (%)
Sex		
Male	228 (75.5)	74 (24.5)
Female	198 (73.9)	70 (26.1)
Age		
2-3 years old	198 (78)	56 (22)
4-5 years old	222 (72.3)	85 (27.7)
Caregivers’ schooling		
>8 years	349 (73.6)	125 (26.4)
≤8 years	77 (80.2)	19 (19.8)
Household income		
A	82 (81.2)	19 (18.8)
B	261 (75.9)	83 (24.1)
C-D	68 (67.3)	33 (32.7)
Dental pain		
No	392 (80.2)	97 (19.8)
Yes	31 (39.7)	47 (60.3)
Dental trauma		
No	217 (77.8)	62 (22.2)
Yes	209 (71.8)	82 (28.2)
Anterior open bite		
Absent	341 (76.1)	107 (23.9)
Present	85 (69.7)	37 (30.3)
Overjet		
Absent	292 (73.4)	106 (26.6)
Present	134 (78.4)	37 (21.6)
dmft		
Absent	342 (85.5)	58 (14.5)
Present	84 (49.4)	86 (50.6)
pufa		
Absent	413 (77.3)	121 (22.7)
Present	13 (36.1)	23 (63.9)

In the adjusted logistic regression model (Table 2), the perception of caregivers of poor oral health status in their children were related to dental pain, dental trauma, anterior open bite, dental caries experience (dmft), and the consequences from dental caries (pufa). The chance of a caregiver considering a child’s oral health to be poor was 3.30 times higher (95%CI 1.84-5.91; p<0.001) when there was a report of dental pain. Regarding clinical oral characteristics, the chance of a caregiver considering a child’s oral health to be poor was 1.68 times higher (95%CI 1.06-2.68; p=0.029) when the child had a history of traumatic dental injury, 1.98 times higher (95%CI: 1.16-3.38; p=0.012) when the child had open bite, 4.92 times higher (95%CI 3.05-7.93; p<0.001) when the child had dental caries, and 3.78 times higher (95%CI 1.63-8.76; p=0.002) when the child had consequences from caries.

**Table 2:** Logistic Regression for the perception of caregivers of their children’s poor oral health and independent variables. Florianópolis, Brazil.

	The perception of caregivers of their children’s oral health					
	Unadjusted			Adjusted*		
	OR	95%CI	p-value	OR	95%CI	p-value
Sex						
Male	1		0.658			
Female	0.92	0.63-1.34				
Age						
1-3 years old	1		0.126	1		0.367
4-5 years old	1.35	0.92-2.00		0.80	0.50-1.30	
Caregivers’ schooling						
>8 years	1		0.178	1		0.721
≤8 years	0.69	0.40-1.19		1.15	0.53-2.47	
Household income						
A	1		0.070	1		0.273
B	1.37	0.79-2.40		1.10	0.53-2.30	
C-D	2.09	1.09-4.01		1.71	0.73-4.03	
Dental pain						
No	1		<0.001			**<0.001**
Yes	6.13	3.70-10.15		3.30	1.84-5.91	
Dental trauma						
No	1		0.102	1		**0.029**
Yes	1.37	0.94-2.01		1.68	1.06-2.68	
Anterior open bite						
Absent	1		0.148	1		**0.012**
Present	1.39	0.89-2.16		1.98	1.16-3.38	
Overjet						
Absent	1		0.209	1		0.337
Present	0.76	0.50-1.17		0.78	0.47-1.30	
dmft						
Absent	1		<0.001	1		**<0.001**
Present	6.04	4.01-9.09		4.92	3.05-7.93	
pufa						
Absent	1		<0.001	1		**0.002**
Present	6.04	2.98-12.28		3.78	1.63-8.76	

*Adjusted by age, parents’ schooling, household income, dental pain, dental trauma, anterior open bite, overjet, decayed, missed, and filled teeth (dmft), and pulp involvement, ulceration, fistula, or abscess (pufa); OR: *Odds Ratio*; 95%CI: 95% confidence interval.

## DISCUSSION

The results of the present study confirm that the perception of caregivers of poor oral health in their children are associated to their children’s oral health status.

A worse perception of children’s oral health increases with the occurrence of dental caries and its consequences, as well as the report of dental pain. The evolution of dental caries, with the worsening of signs and symptoms, such as dental pain, pulp involvement, ulceration, fistula, and abscess, may explain these results, just like demonstrated by previous studies that used the pufa index.^19^ ^,^ ^20^ Negative impacts on quality of life include difficulties in chewing, studying, smiling, playing, or socializing with others, as well as parental distress and affected family functioning, all of which can influence the perception of caregivers regarding their children’s oral health status.^21^ ^,^ ^22^ ^,^ ^23^ ^,^ ^24^

Gomes et al.^5^ also point out the relation between a negative impact on OHRQoL and perceptions regarding their children’s oral health status. Moreover, other authors studying the direct relation between dental caries and perceptions of poor oral health in children report similar results to those described in the present investigation.^5^ ^,^ ^25^ Piovesan et al.^25^ studied 455 children aged one to five years old and their caregivers, and found that the prevalence of perception of a poor oral status was 2.52 times higher among caregivers of children with dental caries than in those whose children were caries-free. Gomes et al.^5^ assessed 843 children aged three to five years old, and found an association between the perception of poor oral health with the interaction of dental caries and dental pain, with the odds of a negative perception being ten times higher in the occurrence of this condition.

The present findings underscore an important aspect: the perception of caregivers of their children’s poor oral health was associated to untreated dental caries and its consequences. This leads to seeking emergency dental care,^26^ increased treatment costs for both caregivers and health services,^27^ development of dental fear/anxiety in children, behavioral problems,^28^ an increased risk of further caries,^29^ and negative impacts on the OHRQoL of children and their caregivers.^21^ ^,^ ^22^ ^,^ ^23^

Open bite and traumatic dental injury were also related to perceptions of children’s poor oral health status. These associations are related to esthetic and functional problems, which exert an impact on their quality of life.^3^ ^,^ ^11^ ^,^ ^30^ In fact, some authors state that the severity of traumatic dental injury in preschoolers is a determinant factor of the negative impact on the OHRQoL.^3^ ^,^ ^30^

Studies on the association between perception of children’s oral health status, malocclusion, and dental trauma offer divergent results.^5^ ^,^ ^25^ A study with preschoolers found no association between perceptions of oral health status and either traumatic dental injury or malocclusion.^5^ Another study found an association to open bite, but not to a traumatic dental injury.^25^ Results related to traumatic dental injury differ from those of the present study, which may be due to geographical and socioeconomic differences. Moreover, failure to classify tooth injuries in terms of severity impedes the estimation of the occurrence of complicated fractures in samples, which could also explain this divergence. Gomes et al.^5^ evaluated all types of malocclusion together, which may explain the failure to find an association.

In the present study, socio-demographic factors were not related to perception of children’s oral health status. Some studies showed different results between the perception of poor oral health with the schooling of caregivers^5^ ^,^ ^7^ and income.^7^ ^,^ ^25^ They were conducted in places that may represent different socioeconomic and geographical conditions compared to the present study. Moreover, these different income-related outcomes may be due to the use of different classification criteria, because Talekar et al.^7^ used the Federal Poverty Line as a classification of USA; Gomes et al.^5^ used a monthly household income as one Brazilian minimum wage; and Piovesan et al.^25^ used a household income as three Brazilian minimum wages. In the present study, the classification criteria were according to the Brazilian Association of Research Companies (Associação Brasileira de Empresas de Pesquisa),^18^ which considers the number of comfort items in households

The present study has limitations to be addressed. The cross-sectional design does not enable establishing causality. Therefore, prospective studies are needed. Moreover, a questionnaire was used, which is subject to the interpretation of the respondent, and responses could be subject to memory bias. Besides that, the sociodemographic factors are limited in caregivers’ schooling and household income, without a more comprehensive assessment of other conditions that could influence the quality of oral health. On the other hand, the positive characteristics were the execution of a pilot project to test the application of the questionnaire and the indexes used to evaluate children’s oral health status, the application of clinical indexes by examiners who had undergone training and calibration exercises and who were blinded to the caregivers’ responses on the questionnaire, sample calculation to ensure the internal validity of data, and the randomized participant selection process. External validity of this study applies to children with the same eligibility criteria. In order to improve this validity, further studies with a random representative sample in different places involving public and private schools must be conducted.

The present findings reveal that the perception of children’s poor oral health occurred when consequences of dental caries were seen, regardless of their caregivers’ income or educational level, because they showed no association to the perception of caregivers. Thus, the focus should be on regular dental appointments to improve caregivers’ knowledge of preventive behaviors and early detection of carious lesions. Hence, despite the increase in the number of visits to the dentist, dental appointments would be faster, involve lower costs and fewer noninvasive treatments, and reduce the risk of developing new caries.^27^ Future prospective studies are needed to investigate the relation between perceptions regarding oral health and the use of health services.

In conclusion, perceptions of poor oral health in children were associated to the occurrence of dental caries, its consequences, reports of dental pain, open bite, and traumatic dental injury. No associations to overjet or socio-demographic factors were found.
